# Coordinate-aware three-dimensional neural network for lower extremity arterial stenosis classification in CT angiography

**DOI:** 10.1016/j.heliyon.2024.e34309

**Published:** 2024-07-09

**Authors:** Chenwei Zhou, Shengnan Cao, Maolin Li

**Affiliations:** a Department of Radiology, Songjiang Hospital Affiliated to Shanghai Jiao Tong University School of Medicine, Shanghai, China; b Department of Radiology, Shanghai TCM - Integrated Hospital, Shanghai University of Traditional Chinese Medicine, Shanghai, China; c Department of Computer Science, City University of London, London, United Kingdom

**Keywords:** Lower extremity arterial disease, Computed tomography angiography, Deep learning, Three-dimensional neural network, CoordConv

## Abstract

**Background:**

Lower Extremity Computed Tomography Angiography (CTA) is an effective non-invasive diagnostic tool for lower extremity artery disease (LEAD). This study aimed to develop an automatic classification model based on a coordinate-aware 3D deep neural network to evaluate the degree of arterial stenosis in lower extremity CTA.

**Methods:**

This retrospective study included 277 patients who underwent lower extremity CTA between May 1, 2017, and August 31, 2023. Radiologists annotated the lower extremity artery segments according to the degree of stenosis, and 12,450 3D patches containing the regions of interest were segmented to construct the dataset. A Coordinate-Aware Three-Dimensional Neural Network was implemented to classify the degree of stenosis of the lower extremity arteries with these patches. Metrics including accuracy, sensitivity, specificity, F1 score, and receiver operating characteristic (ROC) curves were used to evaluate the performance of the proposed model.

**Results:**

The accuracy, F1 score, and area under the ROC curve (AUC) of our proposed model were 93.08 %, 91.96 %, and 99.15 % for the above-knee arteries, and 91.70 %, 89.67 %, and 98.2 % respectively for below-knee arteries. The results of our proposed model exhibited a lead of 4–5% in accuracy score over the 3D baseline model and a lead of more than 10 % over the 2D baseline model.

**Conclusion:**

We successfully implemented a deep learning model, a promising tool for assisting radiologists in evaluating lower extremity arterial stenosis on CT angiography.

## 1 Introduction

Lower Extremity Arterial Disease (LEAD) is characterized by narrowing of the lower extremity arteries, leading to a decrease in arterial blood supply, which causes partial or complete failure of oxygen delivery to the lower extremities and a range of symptoms related to lower extremity ischemia [1], from intermittent pain, numbness, up to gangrenous tissue [2].

LEAD have emerged as a significant public health concern, particularly among the elderly. The overall prevalence of LEAD among individuals aged over 70 is 15 % [3]. Notably, some elderly patients experience rapid progression from asymptomatic to very severe LEAD. Therefore, early diagnosis is crucial for extending the quality of life and lifespan of patients with LEAD.

Lower Extremity Arterial Computed Tomography Angiography (CTA) has been widely used in the non-invasive examination of LEAD [[4], [5], [6]], assisting doctors in analyzing arterial plaque and evaluating the degree of arterial stenosis and occlusion. However, the large data volume of lower extremity CTA and the necessity for 3D reconstruction increase the time required to diagnose correctly.

Deep learning approaches may allow for evaluating lower extremity CTA in a shorter time than human radiologists. The excellent performance of deep learning methods for image analysis indicates that these methods can facilitate the diagnosis of arterial diseases [7,8].

Most existing deep-learning-based computer-aided diagnosis (CAD) models in the cardiovascular system focus on the coronary or vertebrobasilar arteries [[9], [10], [11]]. Only a limited number of studies have focused on peripheral arteries. Furthermore, the diagnostic accuracy of deep learning studies in lower extremity CTA remains unsatisfactory for two reasons. First, most existing models in this domain [12] are trained solely on 2D slices, without utilizing any 3D information from the CTA images. Although some studies [13] have attempted to train neural networks using reconstructed CTA images, such as Maximum Intensity Projection (MIP), these attempts were based on 2D projections of 3D images, fundamentally 2D rather than 3D information. Another potential reason is that most current deep-learning methods do not use the positional information of the region of interest (ROI) in CTA images. For human radiologists, it is important to identify the location of a lesion before analyzing its radiological features. Unfortunately, most existing deep learning approaches fail to teach neural networks the underlying knowledge between an abnormality and its position, which means these neural networks diagnose without knowing where the target is located.

Motivated by these considerations and inspired by the CoordConv algorithm [14], this study introduces the Coordinate-Aware Three-Dimensional Neural Network. This novel approach aims to facilitate the evaluation of lower extremity arterial stenosis on CTA and alleviate the workload of radiologists.

## 2 Methodology

### 2.1 Study population

In this single-center study, we retrospectively analyzed the examinations of patients who underwent lower extremity CTA at Shanghai TCM - Integrated Hospital from 2017 to 2023. The exclusion criteria were: (i) Inadequate enhancement, which compromised the ability to assess the degree of arterial stenosis accurately. (ii) Images exhibiting motion artifacts or any other artifacts that resulted in non-diagnostic quality, specifically those that obscured arterial structures. (iii) Presence of stents in arterial lumens. (iv) Patients with severe calcification that impaired the assessment of arterial stenosis degree, but lacked corresponding Digital Subtraction Angiography (DSA) images as a gold standard reference, were excluded.

### 2.2 Image acquisition

Lower extremity CTA images were acquired using an Aquilion 64 CT scanner (Toshiba Medical Systems, Tokyo, Japan). The following scanning protocols were used: a matrix size of 512 × 512, collimation width of 0.5 × 64 mm, pitch of 0.85, gantry rotation speed of 0.5/rotation, and tube voltage of 120 kV. One hundred milliliters of non-ionic contrast agent (iodixanol, 370 mg/ml) was injected using a high-pressure injector at a flow rate of 4.0 ml/s. The scanning range was from the lower abdominal aorta to below the ankle joint, covering an area of 900–1150 mm. The slice thickness and space between slices were set to 1.00 mm.

### 2.3 Data cropping

To train the neural network more efficiently, we first cropped patches from the bilateral lower extremity arteries in the CTA to build the dataset. For the above-knee arteries, our annotation focused on the superficial femoral, deep femoral and popliteal arteries. For the below-knee arteries, we mainly labeled anterior tibial, posterior tibial, peroneal and foot arteries. The labeler plotted small regions of interest containing segments of the arteries mentioned above in the CTA images. Subsequently, our software automatically cropped 3D patches sized 16 × 16 × 16 pixels from the CTA according to the recorded ROI. Patches were cropped every 50 consecutive slices where the arterial segment was healthy with no stenosis. For segments with plaques, patches including the most severe stenosis were routinely cropped in every segment. In cases of extensive stenosis or obstruction, the number of cropped patches was adjusted based on the length and extent of plaque distribution.

**Fig. 1:**
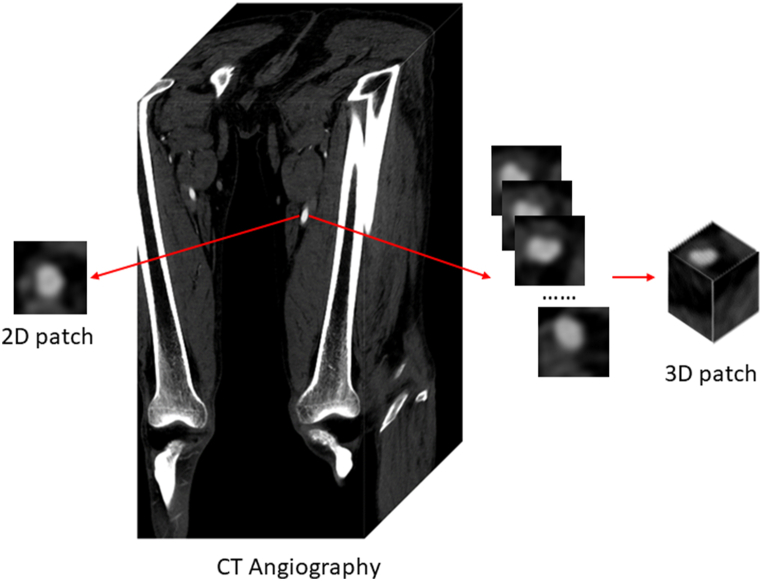
Comparison between 2D and 3D patches.

Unlike other studies that trained networks with only 2D slices or even JPEG instead of DICOM images, our 3D patches retained the spatial information from adjacent slices and raw CT values. Fig. 1 illustrates the differences between the 2D and 3D patches. Notably, the 2D patches used in most previous studies only captured image information from one single slice. By contrast, our 3D patches leverage spatial information across adjacent slices.

### 2.4 Ground truth labeling

The cropped 3D patches were further annotated using the ground truth data to construct the dataset. In this study, the ground truth label of each patch was the degree of lumen stenosis of the lower extremity artery passing through it.

Two experienced radiologists (CZ and SC, with 9 and 8 years of experience in radiology, respectively) evaluated the degree of arterial stenosis by referring to the reconstructed images (MPR, MIP, VR) as well as patients’ DSA examinations (if available). The degree of stenosis was quantified as the percentage of lumen stenosis, defined as the minimal lumen area divided by the reference area. The minimal lumen area was defined as the square of the artery lumen which passed through the patch, and the reference area was defined as the square of a supposed healthy lumen at the same position with no signs of stenosis, calcification, aneurysm or compression. The healthy reference lumen could be inferred from nearby healthy arteries or from observing the corresponding artery on the opposite side.

Owing to the differences in the diameter and evaluation criteria between the above-knee and below-knee arteries, we set different labeling criteria for above and below-knee segments by referring to previous studies [15,16]. In this study, the above-knee artery patches were divided into five groups according to the percentage of lumen stenosis: Group A is with no stenosis (0 %); Group B, mild stenosis (1–50 %); Group C, moderate stenosis (51–75 %); Group D, severe stenosis (76–99 %); and Group E, complete occlusion (100 %). Below-knee arteries were divided into four groups due to their smaller diameter and difficulty in assessing the accurate stenosis: Group A, no stenosis (0 %); Group B, mild stenosis (1–50 %); Group C, moderate or severe stenosis (51–99 %); and Group D, complete occlusion (100 %).

### 2.5 Neural network structure

In this study, our objective was to construct an auxiliary diagnostic model based on a neural network to assist radiologists in evaluating the degree of stenosis in lower extremity arteries. Radiologists only need to tell the neural network the location of interest, and then the system automatically analyzes the artery in the ROI and outputs its degree of stenosis.

To accomplish this, we first construct a neural network capable of handling 3D patches by employing ResNet [17] as the base for our model. ResNet is a widely used high-performance deep learning model that it remains the foundation for many image analysis tasks. While the conventional ResNet was designed for 2D image classification, in this study, we replaced its 2D convolutional cores with 3D cores, extending ResNet to process 3D images [18].

One drawback of these widely used Convolutional Neural Networks (CNN) is their inability to perceive positional information. Most existing CNNs feature a certain degree of translational invariance [19], which means that if the same graphic feature is found in different parts of an image, the neural network's analysis of them is almost the same. However, this feature does not help medical images because normal radiological features and common abnormalities may vary in different parts of the human body. From a medical perspective, existing CNNs, including ordinary 3D CNN, neglect the location information of the target region.

**Fig. 2:**
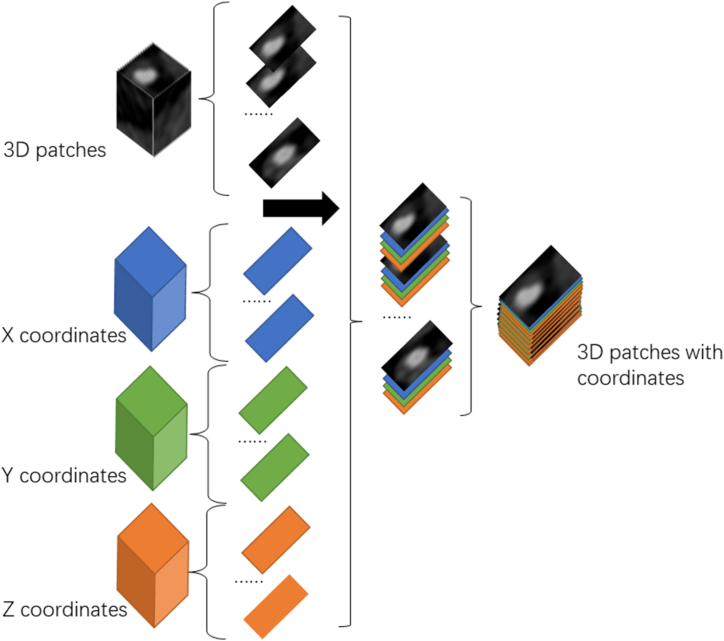
Generating 3D patches with coordinates for the proposed CoordConv model. For a 16 × 16 × 16-pixel CTA patch, x, y and z coordinates were inserted between slices, generating a 16 × 16 × 64 patch that contained CT value and coordinates information.

In this section, we replace traditional convolution with CoordConv to address this issue. CoordConv is an extension of the traditional convolution and is characterized by incorporating the coordinates of each pixel as the input of the neural network. This enables the neural network to learn the relationship between the coordinate information and image features. For the patches we generated in Chapter 2.3, a conventional 3D CNN would directly feed them into the neural network for classification. However, in our CoordConv model, we inserted each pixel's x, y, and z axis coordinates information between adjacent slices. The entire generated patch was fed into the neural network for computation [18]. This process is illustrated in Fig. 2. As a result, the input data of the neural network contained both the CT values and the positional information of each pixel. This approach enables the neural network to autonomously learn the relationship between the degree of stenosis, CT image features, and position of the arteries.

The proposed neural network was designed to process and analyze the aforementioned coordinate-included 3D patches, and output the predicted degree of stenosis. During the training stage, patches from the training set were fed into the neural network and the outputs were used to calculate the loss value with true labels. Backpropagation was then performed to facilitate the learning of the neural network. In terms of the training hyperparameters, we employed Adam optimizer with Cross-Entropy loss function and the initial learning rate was set to 0.001. As the training progressed, the learning rate was dynamically adjusted. Training batch size was set as 1024, and the number of learning epochs was 250.

### 2.6 Dataset split and evaluation metrics

We employed a three-fold cross-validation approach in which the entire dataset was randomly divided into three equal subsets. In each fold, two-thirds of the entire dataset was designated as the training set and subdivided into a training subset (90 %) and a validation subset (10 %). The remaining third was designated as the test set. This process was repeated three times for a comprehensive evaluation to ensure the optimal utilization of all data and avoid random errors.

To better evaluate the performance of our model, baseline 2D and 3D neural networks (without CoordConv) were implemented. A comparative analysis was conducted to evaluated the proposed model's performance against these commonly used baselines. All the training hyperparameters of these comparisons were the same as those in our original model.

During the testing stage, the patches from the testing set were fed into the neural network. The neural network outputted the predicted degree of stenosis, which was then compared with the ground truth annotated by radiologists to evaluate the classification results. To demonstrate the performance of the proposed neural network, the classification accuracy, sensitivity (precision), specificity (recall), and F1 score were used as evaluation metrics, defined as:

$$Accuracy=\frac{TP+TN}{TP+TN+FP+FN}$$

$$Sensitivity=\frac{TP}{TP+FP}$$

$$Specificity=\frac{TP}{TP+FN}$$

$$F1=\frac{2\;*\;Sensitivity\;*\;Specificity\;}{Sensitivity+Specificity\;}$$

Where TP, TN, FP and FN are the number of true positive, true negative, false positive and false negative cases added up within the three-run experiments. The detailed classification results for all classes are listed in the confusion matrices.

Receiver Operating Characteristic (ROC) curves and Area under the Curve (AUC) were obtained by plotting the true positive and false positive rates at different thresholds. A higher ROC curve and larger AUC score indicate a better overall model classification performance. We also employed t-distributed stochastic neighbour embedding (t-SNE) visualization [20] to illustrate the classification results and compare the gaps between models. Instances belonging to the same class are represented by points of the same color on the t-SNE plots. When points of the same color are closely clustered and distant from those of other colors, the model has a strong classification ability.

## 3 Results

Of the 277 patients evaluated, 86 underwent DSA as the golden standard. A total number of 12450 patches were segmented, including 6173 above-knee and 6277 below-knee patches.

Table 1 lists the scores of our coordinate-aware 3D Neural Network and the two comparative models (2D and 3D baseline neural networks). For both above and below-knee segments, our model achieved an accuracy score of over 90 %, indicating a high level of classification performance. Notably, the accuracy, sensitivity, specificity and F1 score of above-knee arteries are all higher than those of below-knee arteries.

**Table 1:** Comparisons of accuracy, sensitivity, specificity and F1 score between different deep learning models on above-knee and below-knee arteries.

Model	2D baseline		3D baseline		Coordinate-Aware	
Position	Above-knee	Below-knee	Above-knee	Below-knee	Above-knee	Below-knee
Accuracy	82.07 %	78.72 %	89.13 %	86.70 %	93.08 %	91.70 %
Sensitivity	78.69 %	75.51 %	87.12 %	84.16 %	92.20 %	90.18 %
Specificity	77.92 %	73.90 %	86.87 %	83.20 %	91.76 %	89.18 %
F1	78.28 %	74.69 %	86.99 %	83.65 %	91.96 %	89.67 %

**Fig. 3:**
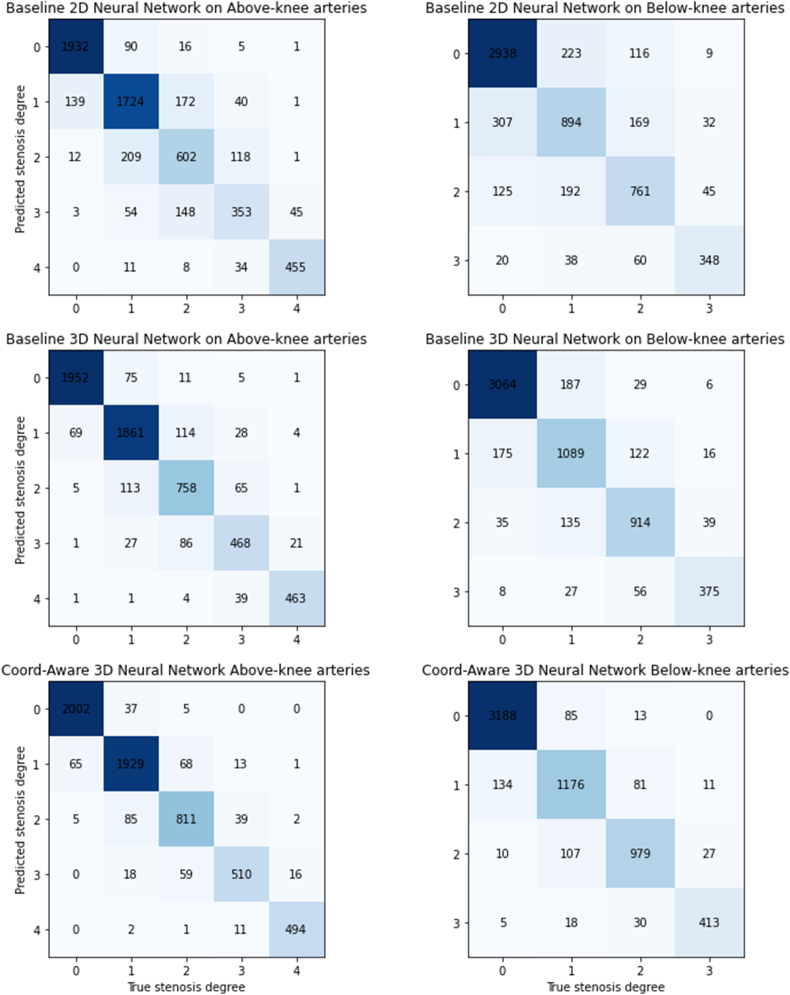
Confusion matrices of different models on above and below-knee arteries. For above-knee arteries, stenosis degrees 0–4 align with group A-E, as defined in Section 2.4. For below-knee arteries, stenosis degrees 0–3 align with group A-D.

Compared with the two baseline models, our proposed model demonstrated obvious superiority, with a 4–5% accuracy score over the 3D baseline model and a lead of more than 10 % over the 2D baseline. More detailed case distributions and classification results are presented in the confusion matrices in Fig. 3.

**Fig. 4:**
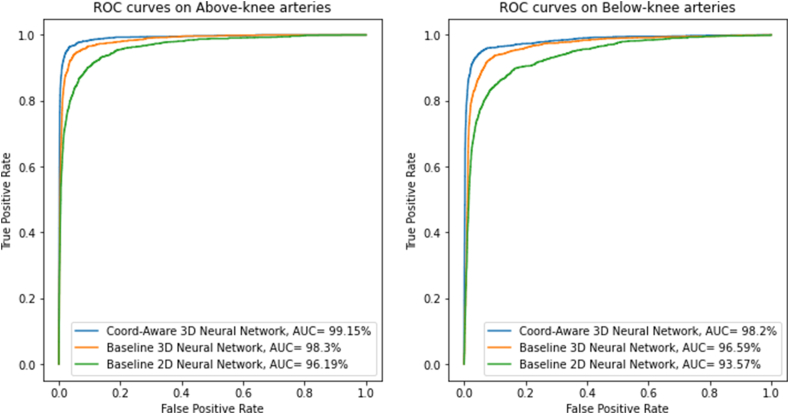
Receiver Operating Characteristic (ROC) curves and area under the curve (AUC) of different models on above and below-knee arteries.

**Fig. 5:**
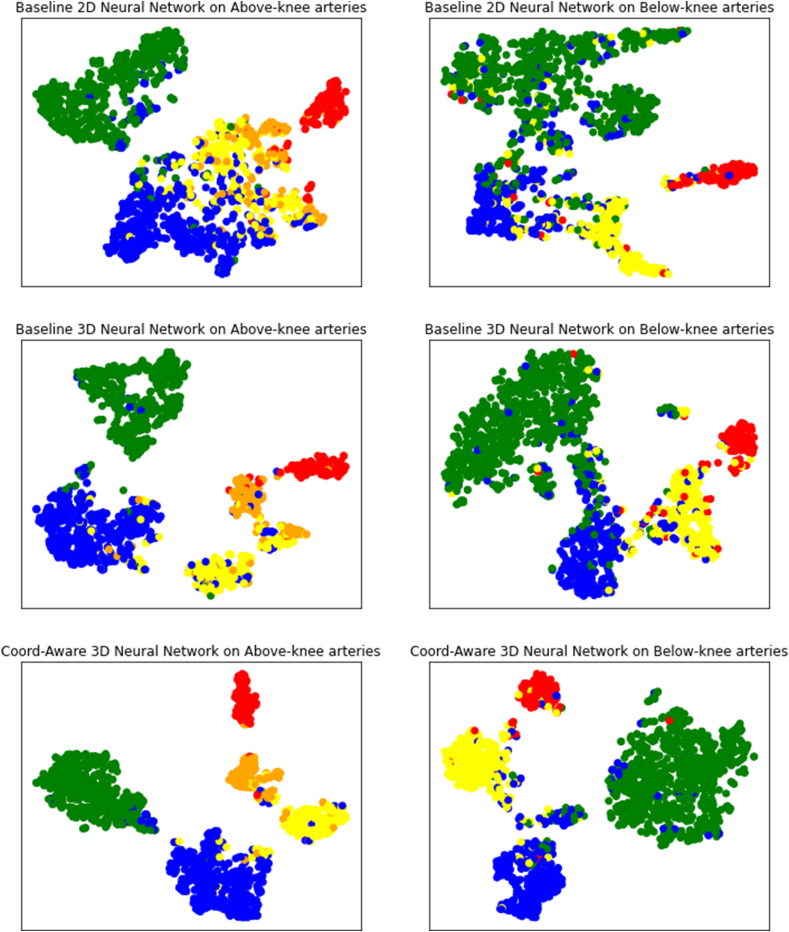
T-SNE visualization plots of the classification results from different models. Stenosis degrees for above-knee arteries: Green: 0 %; Blue: 1–50 %; Yellow: 51–75 %; Orange: 76–99 %; Red: 100 %. For below-knee arteries: Green: 0 %; Blue: 1–50 %; Yellow: 51–99 %; Red: 100 %. (For interpretation of the references to color in this figure legend, the reader is referred to the Web version of this article.)

Fig. 4 shows the results of the ROC analysis. Our proposed model had the highest AUC value, 99.15 % for the above-knee arteries and 98.2 % for the below-knee arteries, significantly higher than those of the two comparative models. The t-SNE visualization plots in Fig. 5 confirm that the proposed model can best discriminate samples belonging to different classes.

## 4 Discussion

Diagnosing lower extremity CTA is a time-consuming task in clinical practice. This research, based on clinical needs and targeting the shortcomings of previous studies, creatively improved upon existing models by referring to radiologists’ workflows.

Our proposed model exhibited two main improvements compared to existing deep-learning models for peripheral CTA. On the one hand, most existing deep learning models employed 2D neural networks, thereby failing to exploit the 3D features from CTA, leading to unsatisfactory diagnostic performance. Accordingly, we employed a 3D neural network to utilize the 3D information from adjacent slices. On the other hand, existing deep learning models based on ordinary CNN neglect the positional information of the target area in CTA scans. We incorporated CoordConv mechanism into our model, allowing the neural network to learn the relationship between CT features and the location the of lower extremity arteries. Quantitative experiments indicated that our improvements effectively enhanced the diagnostic performance of the deep learning model.

**Fig. 6:**
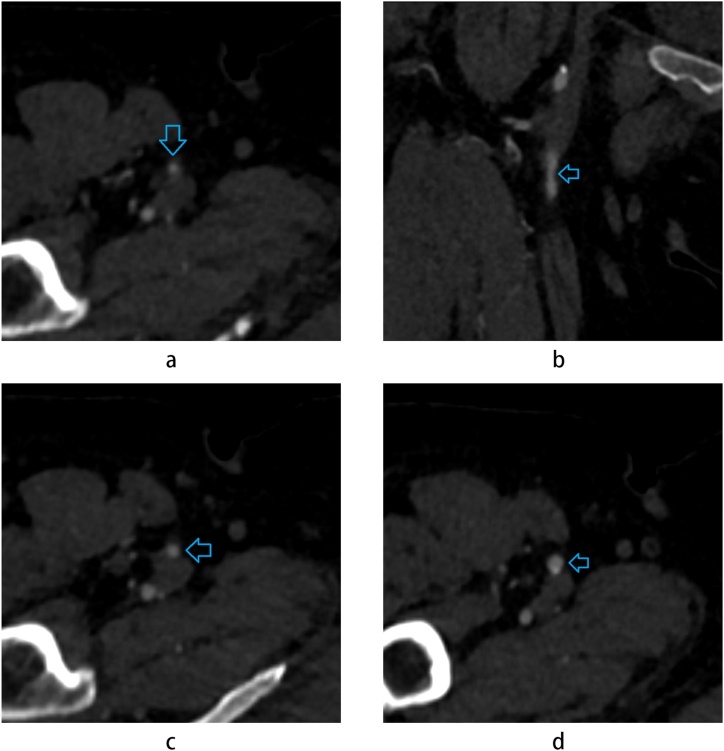
A case in which 2D baseline neural network misdiagnosed. a: cross-sectional slice. b: coronal reconstruction image. c, d: above and below slices.

Real cases also demonstrated that our improved model could correctly classify samples for which existing baseline models fail. For example, Fig. 6 shows how a 3D neural network outperforms a 2D model by exploring adjacent slices. In the cross-sectional slice in Fig. 6 a, it is difficult to correctly judge the degree of stenosis of the superficial femoral artery, as denoted by the blue arrow, leading the 2D neural network to incorrectly classify it as having no stenosis. However, the reconstructed MPR image (Fig. 6 b) and adjacent slices (Fig. 6 c and 6. d) showed signs of mild stenosis in this slice. The 3D neural network utilizes information from adjacent slices and diagnoses correctly.

**Fig. 7:**
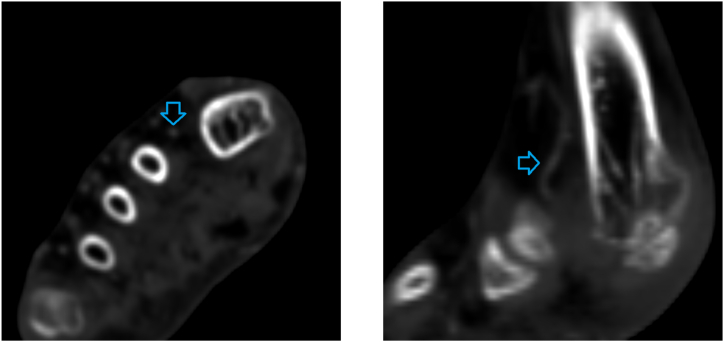
A case in which 3D baseline neural network misdiagnosed.

Compared with an ordinary 3D neural network, our proposed coordinate-aware model can further utilize positional information to improve classification accuracy. As illustrated in Fig. 7, the denoted foot digital artery was misdiagnosed as having mild stenosis by an ordinary 3D neural network due to its small diameter. Our coordinate-aware model learned from training data that it is typical for these arteries to demonstrate small diameters, accurately discerning that there is no stenosis.

The quantitative analysis and sample cases indicated that our model achieved favorable results in classifying lower extremity arterial stenosis. In clinical practice, reconstructing lower extremity CTA and evaluating the degree of arterial stenosis are time-consuming tasks. The deep learning model implemented in this study enabled automatic assessment and achieved high scores on the testing dataset.

**Fig. 8:**
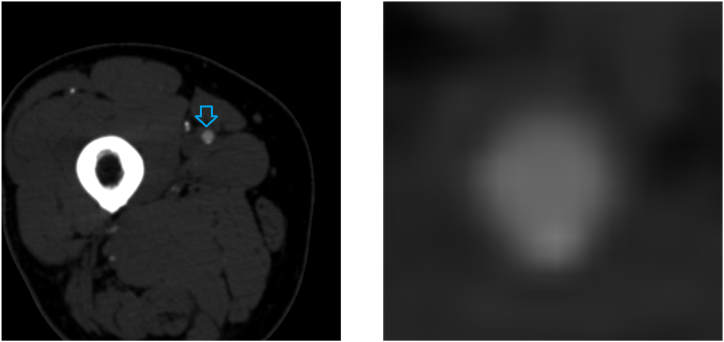
A case in which our proposed model misdiagnosed.

However, in some cases, our algorithm did not produce the correct diagnosis. For example, Fig. 8 shows a cross section of femoral artery, where tiny calcification can be detected along the artery, indicating mild stenosis. Our model failed to identify this microcalcification and incorrectly classified it as no stenosis.

**Fig. 9:**
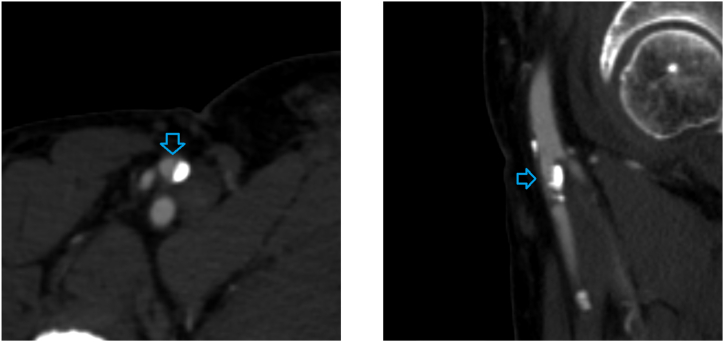
A case in which our proposed model misdiagnosed.

In Fig. 9, the degree of stenosis of the artery indicated by the blue arrow is approximately 50 %, close to the categorical threshold between mild and moderate stenosis. The annotator labeled this patch as mild stenosis, but our model classified it as moderate stenosis. These discrepancies are understandable, but they did reduce the overall performance and provided no help for radiologists.

The above cases indicate one limitation of our research: the diagnostic performance is still imperfect. First, our model exhibited an inadequate diagnostic ability for subtle lesions like microcalcifications. Future work could involve expanding the dataset and constructing a larger neural network. Second, the neural network employed in this study was a classification model, which means that, for cases where the degree of stenosis was at the classification threshold, this model did not provide sufficient help. It may be considerable to build a regression neural network model, which can output numerical stenosis degree predictions to provide radiologists with more accurate information.

Another limitation of this study was its scope. We only attempted to address the issue of classifying the degree of arterial stenosis, while in clinical practice, artery segmentation, and reconstruction are also important and time-consuming. Our proposed model still requires radiologists to tell the location of target arterial segments to the neural network; future work will first explore implementing a segmentation model to find the arteries automatically, then combining it with the proposed stenosis evaluation model. This combination has broad prospects for improving diagnostic ability and simplifying the work of radiologists.

## 5 Conclusion

In summary, this study introduced a Coordinate-Aware Three-Dimensional Neural Network for classifying the degree of arterial stenosis in lower extremity CTA. The experimental results indicated that our proposed model outperformed ordinary 2D and 3D neural networks implemented in existing studies, demonstrating its potential to assist radiologists in the clinical assessment of lower extremity arterial stenosis. Using our model, radiologists only need to locate the target artery, and the system automatically outputs the predicted degree of stenosis.

## Ethical approval

This study was reviewed and approved by IRB of Shanghai TCM-Integrated Hospital with the approval number: 2023-053-1, dated 2023/12/13.

Patient informed consent was exempted by the institution's ethical review board. All data used in this study were anonymized and did not involve personal privacy or commercial interests.

## Funding

No funding

## Data availability

The source code, sample patches and pre-trained checkpoints are accessible on GitHub at: https://github.com/proj4on/coordaware3dstenosis. The CTA images are not publicly available. Access can only be granted upon request to the corresponding author and after approved by the institutional ethics committee.

## CRediT authorship contribution statement

**Chenwei Zhou:** Writing – review & editing, Validation, Resources, Funding acquisition, Data curation, Conceptualization. **Shengnan Cao:** Writing – review & editing, Resources, Data curation. **Maolin Li:** Writing – original draft, Visualization, Software, Project administration, Methodology, Formal analysis.

## Declaration of competing interest

The authors declare that they have no known competing financial interests or personal relationships that could have appeared to influence the work reported in this paper.
